# New York Letter

**Published:** 1880-12

**Authors:** 


### Domestic Correspondence.

### ARTICLE VII.

### NEW YORK LETTER.

*Editors of* CHICAGO MEDICAL JOURNAL AND EXAMINER :

The Cartwright lectures are now being delivered by Professor Roberts Bartholow, of Philadelphia, and the course is deservedly attracting much interest. The subject chosen is the Physiological Antagonism between Medicines; and between Remedies and Diseases. This subject, which has already been worked up, to some extent, by Rutherford, Fothergill and others, will receive much further enlightenment from Bartholow, if one can judge from the lectures already delivered. The first one was devoted to a discussion of the antagonism between opium and belladonna, a point concerning which the lecturer has already written a good deal. The hall was densely crowded and the speaker listened to with great attention. Dr. Bartholow is not an orator at all, but he talks plainly and makes no attempt at rhetoric or elocution. The lecturer is one of the apostles in this country of physiological therapeutics, and he has done more, perhaps, than any one, except Wood, to popularize, so to speak, the scientific method of applying remedies. He lays himself open to the charge of being too ready a generalizer, and too positive in his therapeutical beliefs to satisfy the critical. But such charges, though they weigh against a person on some accounts, are very easily condoned in a teacher of medical students, for whom a good deal of dogmatism is needed.

Therapeutics in this city, by the way, seems to be in a very chaotic condition, if we may judge by what is taught in the medical colleges. In one of these the professor teaches, with great eloquence and earnestness, that all medicines are divided into disease-remedies and symptom-remedies, the former being foreordained from all eternity for the treatment of chronic disease, and the latter for acute. All the students go out from the institution with these and other curious therapeutical ideas packed away in their brains. For the professor creates great enthusiasm among his pupils, as, indeed, he deserves, since, in spite of his idiosyncracies, he is an able man and a magnetic speaker.

In another college, the professor of therapeutics has practically given up entirely the idea of classifying medicines. He simply divides them into mineral and vegetable bodies and then makes subdivisions of the former on a chemical basis. While the first professor referred to is transcendental, and is, in a measure, a teacher of specifics, professor number two is materialistic and a pessimist. In the former's lectures one gets a spirit of hopefulness and leaves with a vague impression that there is a remedy for everything; in the latter's lectures one catches, in undertone, the idea that nobody knows very much about therapeutics anyway. No doubt he is the nearer right.

It is not often that elegant buildings and hospitals are erected for the benefit of medical or surgical specialties. It is only in large cities that the conditions are met which call for or allow such structures. Even in New York, almost all the costly hospitals are general in character. Up to a comparatively recent time this was entirely the case, but of late there seems to be developing a demand for elegant special hospitals. The Orthopædic Hospital is quite a fine building, and is, I believe, the first one of its class in the city. Not long ago the second building of the Woman's Hospital was finished. But the crowning structure of all seems likely to be the Manhattan Eye and Ear Hospital, whose erection is now going on. It is a brick building, with brown-stone basement and trimmings, and is to be five stories high. It will cost nearly a hundred thousand dollars, of which, at last accounts, not a very large part had been raised. The hospital has one particularly good feature in its management, and that is, the union of medical with lay authority on its governing board. That this accounts in part for its prosperity, there can be no doubt. It is a curious fact regarding the three special hospitals which I have just mentioned that they are all grouped near the Grand Central depot, and are consequently in the noisiest and worst part of the city. Close by the Woman's Hospital there is a constant rattle of trains, jangling of bells and screaming of whistles. It might be interesting to try and estimate the therapeutical effect of multitudinous noises upon morbid conditions of the uterus.

The medical activity of New York, as evinced in its various societies, is now at its highest pitch. There are in the city more than a dozen different organizations which are devoted to the advancement of the interests of medical science. The largest one is the County Medical Society, which has the peculiar responsibility of looking after our morals and manners. It is the society with which every regular physician is expected to affiliate. Its membership, however, is probably not over 700, while the total number of medical men registered in the county, under the new law, is nearly 2,500. As there are only between one and two hundred homœopaths in the city, it follows that we have a great many persons here who are either irregular or will not affiliate. Still the society is a powerful and active one.

The New York Academy of Medicine now has the greatest prestige among our medical organizations, for the character of its work and of its members—as well as for the balance which lies in its treasury. It owns an elegant building, a large library, and takes all the medical journals ; and it generously throws open its doors to the whole profession, whether members or not. It is constantly increasing in wealth and membership, and it deserves, by the liberal spirit that is displayed, the reputation which it is gaining.

In the New York Pathological Society there are many persons whose medical and scientific attainments are unsurpassed. This is especially the case as regards the younger part of its members.

The society, as a rule, does most excellent work at meetings. Occasionally this is not the case, however, as happened not long ago. A medical gentleman, from Baltimore, I forget his name, was invited to show to the society the results of his recent investigations into the nature of syphilis. The gentleman in question appeared, and having hedged himself in with an imposing array of microscopes, announced that he had found some peculiar rod-shaped organisms, which were, he believed, the specific germs of syphilis. He believed this, because he had always found these spores in chancres, and also in the indurated glands that accompany or follow the initial lesion of syphilis. The spores were rod-shaped bodies, grouped in a peculiar manner among the cells.

His statements were not received with much favor, as appeared from the ensuing discussion, and it seemed probable to some that the "spores" were nothing but crystals of alum, a substance used in the preparation of the sections. *Quid non sacra fames* of finding a specific germ, compel mortal hearts to do! This interesting exposition was followed by the display of a small fish-bone, with a history of the case and "remarks," the said bone having been extracted with much skill from the upper part of the œsophagus. Then came a discussion over the bone; and then the presentation of a kidney of a man who had suffered from that rare and obscure disease known as chronic diffuse nephritis. But this does not by any means represent what the Pathological Society can do, or generally does.

The Neurological Society has some very active and wide-awake members, and its meetings are almost always made interesting by lively discussions. For some time past it has turned its energies upon psychiatry and the insane asylums. This has resulted in much notoriety for the asylums and a good deal of newspaper prominence for the society. The prospects of asylum reform are, I may say now, considerably brighter than they have been. A committee appointed by the legislature last winter is investigating the State institutions for the insane, and there is promise that it will do its work thoroughly. The system of caring for our State insane undoubtedly needs an overhauling and readjustment, though whether anything very radical can be done under the present political methods, is doubtful.

To return to the societies—New York's wealthiest and most "aristocratic" medical organization is probably the Obstetrical Society, which includes the gynæcologists. This is partly social in its objects, and, after discussing trachelorrhaphy, etc., it then, as is rumored, addresses itself to champagne and oysters with much earnestness. Trachelorrhaphy is indeed a dry subject, compared with champagne.

The Clinical Society, composed for the most part of young men, and very clever ones, is an organization that does some very good work. A paper was read before it a while ago by Dr. F. H. Bosworth on the subject of the effect of tobacco smoking upon the throat. It was the opinion of the reader—an opinion confirmed in the subsequent discussion—that smoking did not affect the throat at all, as a rule, and that genuine smoker's sore-throat is rather a myth. Some exception was made to the innocuousness of cigarettes; and it was, of course, admitted that hard smoking might aggravate a chronic catarrh brought on by other causes.

There is, after all, but a small proportion of the profession that attends with any regularity the meetings of the societies. And there is a very good reason for this. Although occasionally there are excellent papers and discussions, this is not the rule, and whether the paper is good or not there is a large amount of talk that is simply inane following it. Societies are haunted by a few persons for whom age or accident secure respectful hearing, and who insist on using their privileges of the floor, whether they have anything to say or not. Now it is not profitable to listen to discussions upon the value of whale-oil in pleurisies, for instance. Nor can one be aroused to enthusiasm by a two-hour oration on the habits of the tape-worm. Society meetings illustrate the curious physiological fact that when any accident, prenatal or otherwise, arrests the development of the cerebral mass, the posterior part of the third left frontal convolution generally remains intact, or may even be the seat of an extra molecular activity. There are a certain number of persons, generally, but not always in the decline of life, who will get up and talk, whatever may be the subject, and one could easily experience the idea of eternity by listening to them, we will say for a day. Sometimes it is amusing to note the contact between the old school of medical thought and the new, with its display of scientific refinements. A venerable physician of wide reputation, but of somewhat empirical habit of mind, was nearly paralyzed, the other evening, by a bright young neurologist who asked him "if there was not a condition of cerebro-spinal depression in his patient, which contra-indicated the use of opium."

On the whole, a person is hardly to be blamed for staying away from most society meetings, since he runs an excellent chance of being bored, and since what is best in the proceedings can be found later in the medical journals. This is, of course, only one way to look at the matter. From a higher stand-point it might be considered a duty to go and do some work to make the meetings better. Furthermore, there is no doubt that if a New York medical society sets out to have a good meeting, as occasionally happens, there can be nothing more instructive.

Some attempts have been made of late to stir up the subject of veterinary, or comparative, medicine. It is said that there is a great need of educated veterinarians, and that whoever has the courage to encounter the obloquy of being called a "horse-doctor," is sure to earn a handsome income, while, if he has true inclination, he can win a scientific reputation at the same time. The veterinarians of this city easily gain an income of $2,000, and from that upwards. There is a great deal still to be worked up in the diseases of the lower animals. The above are the statements made by Dr. Bates, the Dean of the Columbia Veterinary College of this city. The recent prevalence of pleuro-pneumonia, the discussions upon trichiniasis, bovine tuberculosis, chicken-cholera, anthrax and splenic fever have tended to give a new prominence to the value of comparative medicine, and it is a prominence which is unquestionably deserved.

Our night medical service continues to receive encomiums from various quarters. There were, I believe, about thirty-two calls during October. The Academy of Medicine has passed a vote of thanks to its originator, and thus reaffirmed its indorsement of the project. It does work very nicely—in a way. One of these ways is as follows: when a poor patient calls up a member of the service in the night, he tells him to go to the police-station and ask the officer there to send for him. The doctor thus insures his three dollars. It is quite possible for some permanent arrangement between physician and patient to be made in this way. Thus the system is liable to great abuse, though it would be unfair to say that it has not been properly carried on up to the present time. I noticed that in Paris the expense of the service has of late increased so rapidly that some investigation has had to be made.

We have the divine Bernhardt with us just now. Her acting excites the greatest interest among all; but her attenuation seems to have especially attracted the observation of medical men. Among these, she is reported to be so very thin that whenever she takes a pill, she looks as though she were pregnant!

NEW YORK CITY, Nov. 16, 1880.

---

A DANGEROUS TOY.—At the last séance of the Société d'Hygiène, Dr. Gorecki exhibited one of the little toys resembling a swallow, constructed by supporting a small helix of zinc on a handle provided with an apparatus for giving it a rotary motion like a top. In appearance nothing could be more inoffensive than this simple toy, but in reality it is quite dangerous, especially if the child who plays with it, sets it in motion while the apparatus is held over the head.

One month before, a child eight years old was brought to his eye clinic, who, in spite of the protests of his schoolmasters, had not only played with the toy, but instead of holding it at a distance from his body with the arms extended when it was set in motion, had persisted in holding it directly above his face, in order to secure more force in propelling it. When the zinc helix flew off, it divided the eye in a horizontal line as completely and as cleanly as if the incision had been made for a cataract operation. The aqueous humor, iris, crystalline lens and vitreous body all escaped from the wound, while the retina, released from its position by a severe hæmorrhage, stuck to the posterior face of the cornea. The eye was completely lost. A similar accident is said to have resulted from the impulse given to a playing-card in what is known as "prestidigitation."—*Le Praticien.*

